# Dynamic Conformations of Nucleophosmin (NPM1) at a Key Monomer-Monomer Interface Affect Oligomer Stability and Interactions with Granzyme B

**DOI:** 10.1371/journal.pone.0115062

**Published:** 2014-12-09

**Authors:** Wei D. Duan-Porter, Virgil L. Woods, Kimberly D. Maurer, Sheng Li, Antony Rosen

**Affiliations:** 1 Durham VA Medical Center and Duke University Medical Center, 508 Fulton St, Durham, NC, 27705, United States of America; 2 University of California San Diego, 9500 Gilman Drive, Room 4011, La Jolla, CA, 92093-0656, United States of America; 3 Johns Hopkins University School of Medicine, 5200 Eastern Avenue, Mason F. Lord Building, Center Tower, Suite 4200, Baltimore, MD, 21224, United States of America; University Medical Center Utrecht, Netherlands

## Abstract

Nucleophosmin (NPM1) is an abundant, nucleolar tumor antigen with important roles in cell proliferation and putative contributions to oncogenesis. Wild-type NPM1 forms pentameric oligomers through interactions at the amino-terminal core domain. A truncated form of NPM1 found in some hepatocellular carcinoma tissue formed an unusually stable oligomer and showed increased susceptibility to cleavage by granzyme B. Initiation of translation at the seventh methionine generated a protein (M7-NPM) that shared all these properties. We used deuterium exchange mass spectrometry (DXMS) to perform a detailed structural analysis of wild-type NPM1 and M7-NPM, and found dynamic conformational shifts or local “unfolding” at a specific monomer-monomer interface which included the β-hairpin “latch.” We tested the importance of interactions at the β-hairpin “latch” by replacing a conserved tyrosine in the middle of the β-hairpin loop with glutamic acid, generating Y67E-NPM. Y67E-NPM did not form stable oligomers and further, prevented wild-type NPM1 oligomerization in a dominant-negative fashion, supporting the critical role of the β-hairpin “latch” in monomer-monomer interactions. Also, we show preferential cleavage by granzyme B at one of two available aspartates (either D161 or D122) in M7-NPM and Y67E-NPM, whereas wild-type NPM1 was cleaved at both sites. Thus, we observed a correlation between the propensity to form oligomers and granzyme B cleavage site selection in nucleophosmin proteins, suggesting that a small change at an important monomer-monomer interface can affect conformational shifts and impact protein-protein interactions.

## Introduction

Nucleophosmin (NPM1)/B23 is an abundant, nucleolar autoantigen and tumor antigen that is over-expressed in rapidly proliferating cells [1]–[5]. The wild-type protein is required for normal proliferation and differentiation [1], [4]–[7], and has multiple attributed functions, including transcriptional stimulation [8]–[10], nucleic acid binding and chaperone roles [11]–[14], and interactions with p53/p14^ARF^ pathways [15]–[20]. NPM1 has been defined as an autoantigen in systemic lupus erythematosus (SLE) [21], scleroderma [22], and hepatocellular carcinoma (HCC) [23], [24]. Structurally, NPM1 belongs to the nucleoplasmin (NLP) family of proteins, and forms pentamers in a ring-like configuration [25]–[30]. In NLP family members, pentamer formation requires a highly similar amino-terminal region, known as the core oligomerization domain [25], [28], [31]. The available X-ray crystal models of NPM1, like those for other NLP members, do not contain the carboxyl-terminal domains [29], [30].

In previous studies, Ulanet et al. [32] showed that NPM1 was not only over-expressed in HCC tissue when compared with non-malignant liver cells, but also had several distinct biochemical properties. When compared with surrounding cirrhotic tissue of the same specimen and normal non-cirrhotic livers, NPM1 in tumor tissue had increased mobility by sodium dodecyl sulfate polyacrylamide gel electrophoresis (SDS-PAGE), and also exhibited an additional form, consistent with a high molecular weight, SDS-stable oligomeric complex [32]. Furthermore, NPM1 in HCC cells was more sensitive to granzyme B cleavage [32], a property enriched among human autoantigens as compared with non-antigen proteins [33], [34]. In attempting to determine the biochemical basis for these observed differences, Ulanet et al. [32] found that a construct modeling alternative initiation at the seventh methionine, M7-NPM, had identical features to the tumor form of NPM1 described above. Interestingly, since our initial studies, alternative initiation of translation at the fifth and ninth methionines in mouse and human NPM1 have been identified by other groups, using high resolution ribosome profiling and amino-terminal peptide proteomics [35]–[37]. Although initiation at the seventh methionine was not found in these studies, it remains possible that M7-NPM may occur in specific cell types not included in the above experiments, including pre-cancerous or malignant cells. Additionally, M7-NPM may share similar biochemical and structural properties with constructs lacking the first four or eight amino-terminal residues, as would occur with translational initiation at the fifth or ninth methionines, respectively.

In this study, we have analyzed the dynamic conformations of wild-type NPM1 and M7-NPM using deuterium exchange mass spectrometry (DXMS), in order to better understand the structural basis for altered oligomer formation. DXMS has been used to study the conformational changes of proteins under various conditions and in combination with a multitude of binding partners and cofactors [38]–[43]. This technique measures the exchange of back-bone amide protons for solvent deuterons, and with the aid of protease digestion, maps the accessibility of various regions with peptide-level resolution; in turn, the presence of exchange captured under various conditions often indicates very specific local and global structures. Furthermore, the morphology of the deuterated peptide spectra is determined by both 1) local interactions which affect the accessibility of amide protons, and 2) the global and regional structures which determine the kinetics of catalytic deuteration by hydroxyl ions [38]–[40], [44]–[47]. The relative kinetics of these multiple processes produce distinct DXMS data. For example, “unfolding” proteins by increasing temperature or using higher concentrations of denaturant generate bimodal mass spectra that are classically termed EX1; these data reflect local “refolding” rates which are much smaller than rates of catalytic deuteration [39], [47]–[49].

Here, we describe the unexpected discovery of EX1 kinetics at a key monomer-monomer interface that includes the β-hairpin loop, indicating significant local structural flexibility in wild-type NPM1 under non-denaturing conditions. This interface was an area of important differences between wild-type NPM1 and M7-NPM structure, and furthermore, targeted disruption of part of this interface, at the β-hairpin “latch”, prevented formation of stabilized oligomers. Finally, mutations that affect nucleophosmin oligomer formation also changed recognition and cleavage by granzyme B. Thus, we present evidence of dynamic structural shifts in NPM1 that significantly impact protein-protein interactions and may represent a target for altering NPM1 function.

## Materials and Methods

### Cloning, mutagenesis, and ^35^S-methionine-labeled in vitro transcription and translation (IVTT)

For expression of untagged recombinant wild-type NPM1, cDNA for the long form of wild-type human NPM1 (isoform 1, accession NM_002520) was amplified using primers 5′-gagaccatggaagattcgatggacatggac-3′ and 5′-taactaagcttttaaagagacttcctccactgc-3′, digested with NcoI (amino-terminus) and HindIII (carboxy-terminus), before ligation with the similarly digested pET-28a(+) vector (Novagen). This placement led to removal of the amino-terminal poly-histidine and T7 tags, while an intact stop codon prevented translation of the carboxyl-terminal poly-histidine tag. M7-NPM was similarly cloned, but with primer 5′-gagaccatggacatgagccccctgagg-3′ instead of the wild-type 5′ primer, to create a truncated protein beginning at the seventh methionine.

Y67E-NPM was made from wild-type NPM1 plasmids using primers 5′-gaagcagaggcaatgaatgaggaaggcagtccaattaaag-3′ and 5′-ctttaattggactgccttcctcattcattgcctctgcttc-3′, according to the QuikChange site-directed mutagenesis kit (Stratagene). However, we substituted Platinum Pfx DNA polymerase (Invitrogen) and XL2-Blue Ultracompetent cells (Stratagene) for the reagents indicated in the original kit.

To generate myc-tagged proteins, nucleophosmin constructs were amplified with 5′ primers containing EcoRI sites (NPM1 and Y67E-NPM with 5′-tgccgcgaattcatggaagat tcgatggacatgg-3′, M7-NPM with 5′-tgccgcgaattcaccatggacatgagccccctgagg-3′), and a 3′ primer with NotI site for in-frame translation of myc-tag sequence (5′-gagagcggccgcaagagacttcctccactgccagagatc-3′). Constructs and pcDNA3.1-myc vector were then digested with EcoRI and NotI, before ligation and transformation into DH5α cells for propagation of plasmids.

IVTT products were made using various cDNAs in pcDNA3.1 vectors (Invitrogen), ^35^S-radiolabeled methionine, and TNT T7 Coupled Reticulocyte Lysate System (Promega). For testing the ability of mutants to form oligomeric complexes with wild-type NPM1, untagged NPM1 cDNA was mixed with 25, 50 or 100 ng of myc-tagged wild-type NPM1, M7-NPM or Y67E-NPM cDNA before adding reagent mix for IVTT (12.5 µl final reaction volume for each). These reactions were incubated at 30°C for 100 mins before termination with gel application buffer (2% SDS final); products were analyzed by 8% SDS-PAGE and detected with autoradiography.

### Purification of recombinant wild-type NPM1 and M7-NPM

Expression cultures were inoculated 1∶100 with overnight starter cultures of freshly transformed BL21 (DE3) pLysS cells (Novagen). Cells were grown in Terrific Broth (TB) until A_600_ of 1.0 or higher, then induced with 1 mM isopropyl-beta-D-thiogalactopyranoside (IPTG) for 6 hours at 30°C. After expression, bacterial pellets were centrifuged 10 minutes at 5200×g and 4°C, washed once with 50 mM Tris-Cl, pH 7.9, and centrifuged again as previously. Pellets were stored overnight or longer at 80°C, then thawed on ice for 20–30 minutes and resuspended in wash buffer (50 mM Tris-Cl, pH 7.9, 5 mM MgCl_2_, 0.1 mM EDTA, pH 8.0, 0.1 mM DTT, 10% glycerol) with 0.1 mM phenylmethylsulphonyl fluoride (PMSF) and lysozyme at 40 µg/ml. Cells were sonicated 10–30 seconds at 4°C, DNase I was added at 20 µg/ml, and cell suspensions were spun for 1 hour at 19010×g (maximum force for the Sorvall SL-1500 rotor used) before 0.45 µm filtration (EMD Millipore Steriflip system).

Wash buffer with 1 M (NH_4_)_2_SO_4_ was used to bring the filtered supernatant to 100–140 mM (NH_4_)_2_SO_4_ before loading on a heparin affinity column (HiTrap family, Amersham Biosciences/GE Healthcare). Elution of both wild-type NPM1 and M7-NPM first appeared with 200 mM (NH_4_)_2_SO_4_, and was complete in a rapid gradient to 1 M (NH_4_)_2_SO_4_. Relevant fractions were concentrated using Centriplus YM-30 (Millipore) and dialyzed into wash buffer overnight at 4°C.

We next applied the dialyzed elution samples onto the strong anion-exchange column, Q XL (HiTrap family, Amersham Biosciences/GE Healthcare) and washed with 300 mM KCl before elution with 480 mM KCl (all salt solutions in wash buffer). Appropriate fractions containing nucleophosmin proteins were concentrated and dialyzed as noted above, and stored at −80°C. Samples were then thawed on ice for additional gel filtration (HiPrep 26/60 S-300 HR column, Amersham Biosciences/GEHealthcare) and fractions from a single eluting peak were collected and concentrated using Centriplus YM-100 (EMD Millipore). All recombinant protein stocks were stored at −80°C, in wash buffer with 150 mM KCl.

Final yields for this 3-step purification were 2–3 mg for initial 2-liter bacterial cultures. Protein samples from multiple purifications were very similar in composition, and immunoblots with polyclonal rabbit antiserum (α-NPM) identified monomer and oligomer bands, as well as degradation products. Initial samples of wild-type NPM1 and M7-NPM were confirmed by amino-terminal sequencing for five cycles, before being used for the first round of DXMS studies. Protein concentrations were confirmed using colorimetric protein assay (BioRad) with bovine serum albumin as the standard.

### DXMS analysis

Wild-type NPM1 and M7-NPM were each concentrated to 8–9 mg/ml in wash buffer (50 mM Tris-Cl, pH 7.9 and 150 mM KCl), before being mixed 1∶3 with non-deuterated or deuterated water with 150 mM KCl (75% deuterated solvent final); non-deuterating reactions were quenched after 1 minute, whereas samples in deuterated buffers were incubated for 10, 30, 100 or 600 seconds at either 4°C or 25°C, before exchange reactions were quenched. We tested various quenching solutions with increasing guanidium hydrochloride (GuHCl) concentrations on non-deuterated samples and found 3.2 M in 0.8% formic acid (1.9 M GuHCl final concentration) produced fragments with the best overall coverage and resolution. Thus, we used 3.2 M GuHCl in 0.8% formic acid to quench all subsequent deuteration reactions. Deuterated samples were immediately aliquoted into glass vials, and frozen on dry ice; duplicate or triplicate samples were prepared for each reaction condition, with 40–45 µg protein in each vial. Additionally, equilibrium or “fully” deuterated reactions were carried out by exposing protein samples to 0.8% formic acid in deuterated water overnight, before reactions were stopped with 0.5% formic acid and 3.2 M GuHCl.

All DXMS samples were stored at −80°C at least overnight, before fragmentation and mass spectrometry analysis at the DXMS facility at the University of California, San Diego. The procedure has been described in detail elsewhere [38], [40]; briefly, our samples were subjected to proteolysis by pepsin and *Aspergillus saitoi* fungal protease type XIII, before separation by C18 high performance liquid chromatography (HPLC). The proteases used in our experiments are standard for DXMS and were chosen due to their nonspecific activity (and thus increased likelihood for broad coverage by peptides), and optimum activity under the conditions required to minimize hydrogen exchange (i.e. pH 2–3 and temperatures close to 0°C) [38], [50], [51]. After proteolysis, peptide fragments were injected simultaneously into quadrupole time-of-flight (QTOF) and electrospray ion trap tandem (ESI-MS/MS) spectrometers in the initial optimization experiments, or QTOF alone in later experiments.

In addition to incubation at 4°C with deuterated solutions for the second round of DXMS studies, we also changed the sample buffer to 10 mM Tris-Cl, pH 7.4, with no added salt, and altered the quenching solution to include 16.6% glycerol (about 10% final concentration). Fragments were first identified using Sequest (Thermo Finnigan) and then confirmed and analyzed with proprietary DXMS software [40].

### Visualization of NPM1, NLP and NO38 structures

Solved crystal structures [26]–[29] were visualized and prepared for figures using DeepView Swiss-Pdbviewer (http://swissmodel.expasy.org/spdbv). Protein structure models were found in the Protein Data Bank (currently hosted at http://www.rcsb.org) by full names and/or standard abbreviations (i.e. human nucleophosmin or NPM1, *Xenopus* NO38, nucleoplasmin or NLP, and *Drosophila* nucleoplasmin-like protein or dNLP).

### Granzyme B reactions

IVTT product were diluted in NP-40 lysis buffer (1% Nonidet P-40, 20 mM Tris-Cl, 150 mM NaCl, 10 mM EDTA, pH 7) and purified granzyme B (gift from N. Thornberry, Merck) was added to specified concentrations (usually less than 540 nM). Samples were incubated at 37°C for 1 hour and SDS-PAGE sample buffer (final 2% SDS, 4% glycerol, 40 mM Tris-Cl, pH 6.8) was added immediately. All samples were boiled for 5 minutes, separated by SDS-PAGE and visualized by autoradiography.

## Results

### DXMS analysis of wild-type NPM1 and M7-NPM show similar deuteration patterns

**Figure 1:**
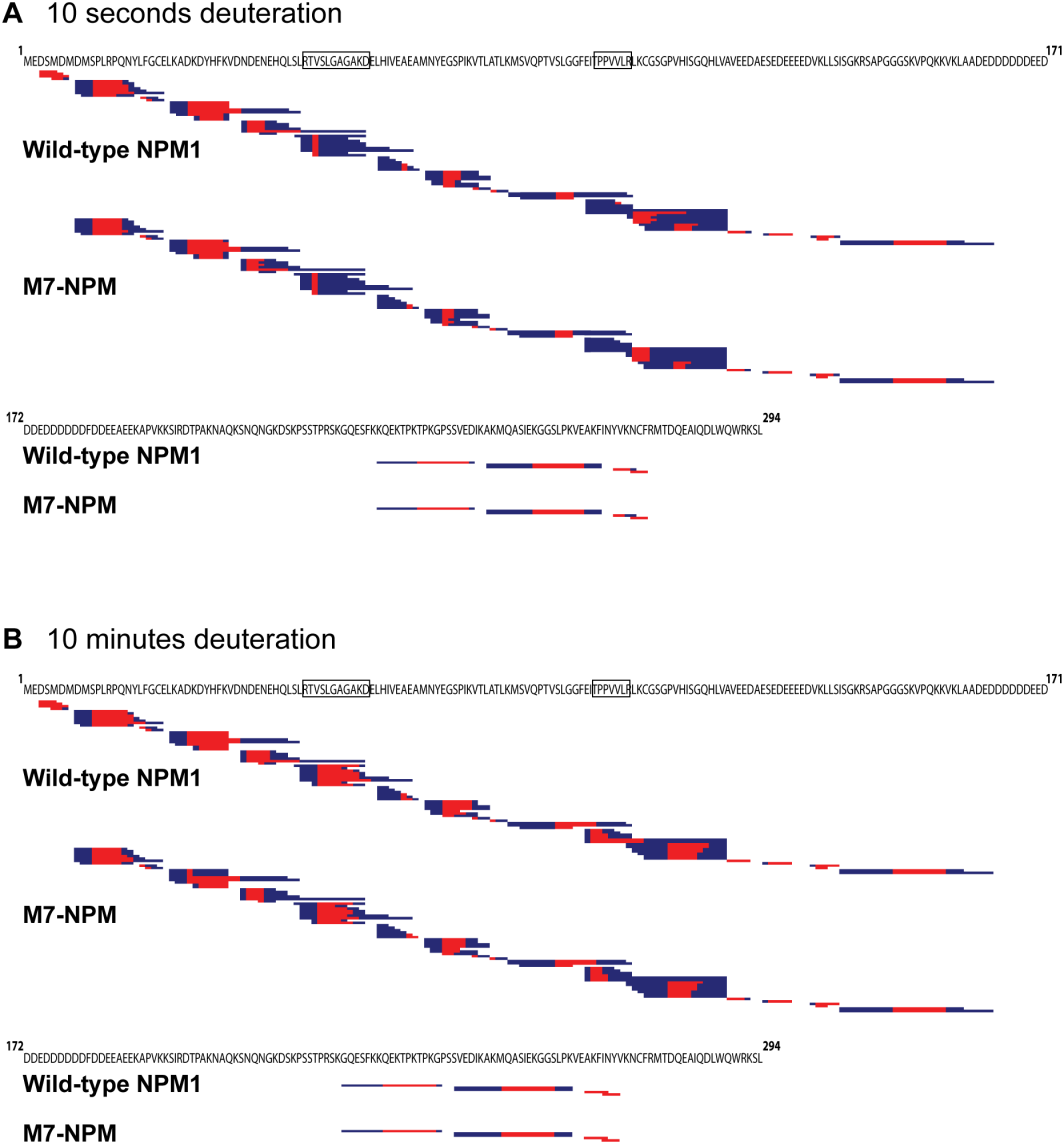
Wild-type NPM1 and M7-NPM are similarly deuterated at room temperature. Wild-type NPM1 and M7-NPM were exposed to 75% deuterated buffer for 10 seconds (A) or 10 minutes (B) at room temperature, before fragmentation with proteases and detection by QTOF. The wild-type protein sequence is represented in two parts; peptides are aligned with wild-type sequence and shown individually as blue bands with red areas. Red regions indicate best guess estimates of deuterated residues based on data from multiple peptides. Boxes around residues in the wild-type sequence highlight regions where both wild-type and M7-NPM peptides showed increased deuteration, with longer incubation times.

We undertook DXMS analysis of recombinant wild-type NPM1 and M7-NPM under non-denaturing conditions at 25°C, in order to determine the structural basis for previously noted biochemical differences. We found similar coverage for both wild-type NPM1 and M7-NPM–identified peptides represented approximately 85% of the amino-terminal region up to residue 170 (numbering according to wild-type NPM1) in the second acidic region, but less than 50% coverage was found for the carboxyl-terminal half (Fig. 1; see S1 File for detailed peptide data). There were minor differences in fragmentation patterns and peptides between wild-type NPM1 and M7-NPM, including the expected absence of the first six residues in M7-NPM samples. Surprisingly, we found that the deuteration patterns for wild-type NPM1 and M7-NPM were largely similar at all incubation times (10 seconds and 10 minutes data shown in Fig. 1). There were two regions in both wild-type NPM1 and M7-NPM proteins which clearly demonstrated increased deuteration with prolonged exposure to solvent, namely residues 45–55 and 96–102 (areas denoted by boxes on the wild-type NPM1 sequence in Fig. 1). The area between these two regions also had increased deuteration with longer incubation, but to a lesser degree (Fig. 1). Finally, the amino-terminal area preceding residue 45 was mostly deuterated in both wild-type NPM1 and M7-NPM after incubation for just 10 seconds (Fig. 1).

### Wild-type NPM1 display dynamic conformations at a specific monomer-monomer interface, including the β-hairpin loop

**Figure 2:**
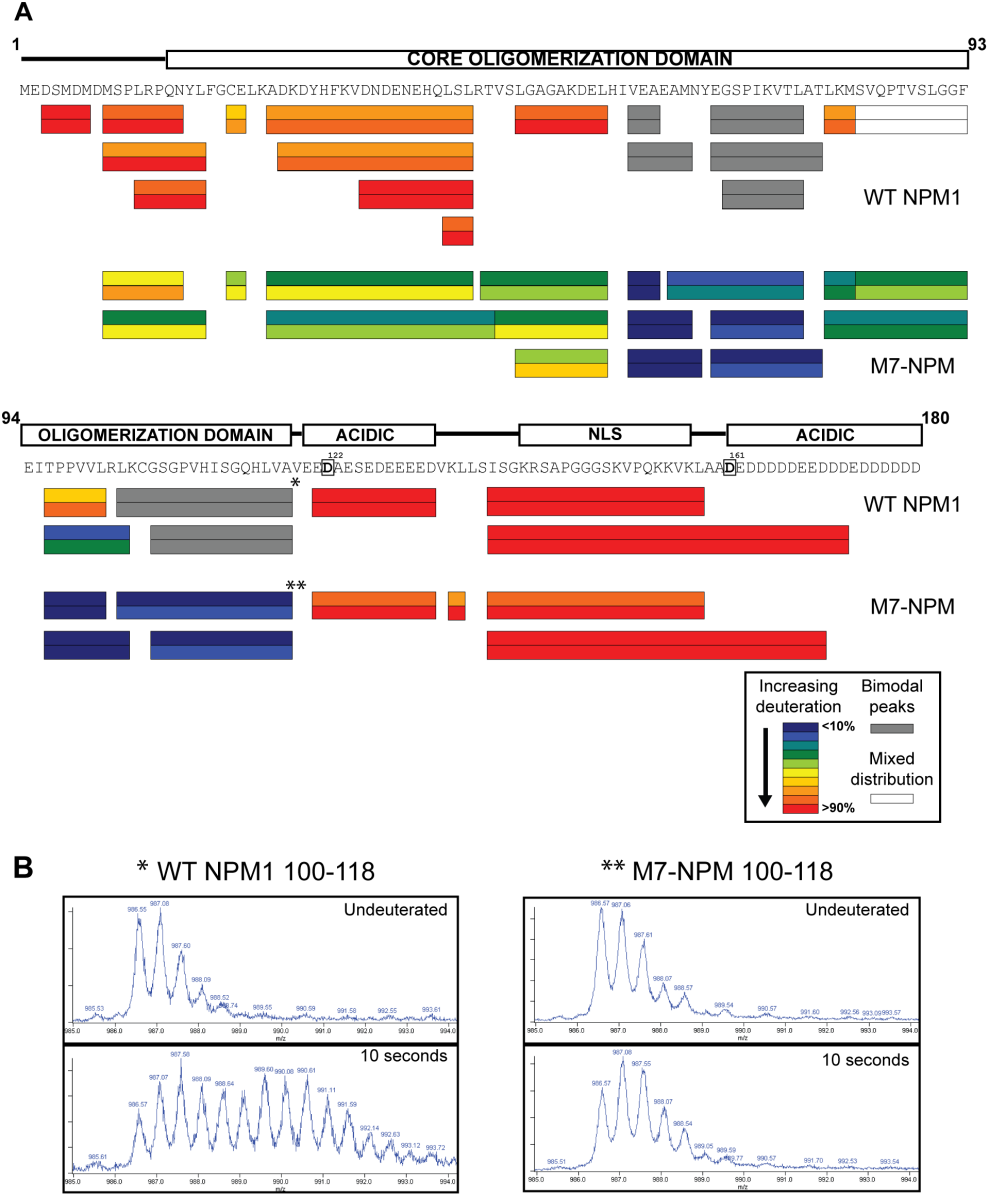
Wild-type NPM1 is more accessible to solvent and displays EX1 deuteration kinetics in several areas. A) Comparison of deuteration patterns for the amino-terminal halves of wild-type NPM1 and M7-NPM, with amino acid sequence for wild-type NPM1 shown below schematic of key domains. Deuteration data for each peptide depicted as two bands of color, the upper and lower ones being percentage deuteration at 4°C after 10 and 30 seconds, respectively. Granzyme B cleavage sites are denoted by bold residues within boxes. Asterisks indicate peptides with mass spectra shown in (B). B) Example of bimodal spectra typical of EX1 kinetics seen for several wild-type peptides, data shown for residues 100–118 of wild-type NPM1 and M7-NPM.

In order to better resolve differences between wild-type NPM1 and M7-NPM, we attempted to reduce deuteration kinetics by decreasing the incubation temperature from 25°C to 4°C. We also increased the acidity of the Tris-Cl buffer from pH 7.9 to 7.4 at 25°C, which maintained the same concentration of hydroxyl ions at 4°C due to the temperature related changes in pH of Tris-Cl buffers. Additionally, we reduced the ionic strength by removing KCl from the sample buffers (salt concentration has been shown to affect NPM1 oligomer stability [30], [52]). Under these new conditions, we found similar peptide coverage, representing greater than 80% of the amino-terminal 180 residues for both wild-type NPM1 and M7-NPM (Fig. 2A; see S1 File for detailed peptide data). However, we detected significantly less deuteration of M7-NPM within the oligomerization domain, whereas wild-type NPM1 peptides in this region either demonstrated extensive deuteration after just 10 seconds, or had bimodal peptide spectra (Fig. 2A).

**Figure 3:**
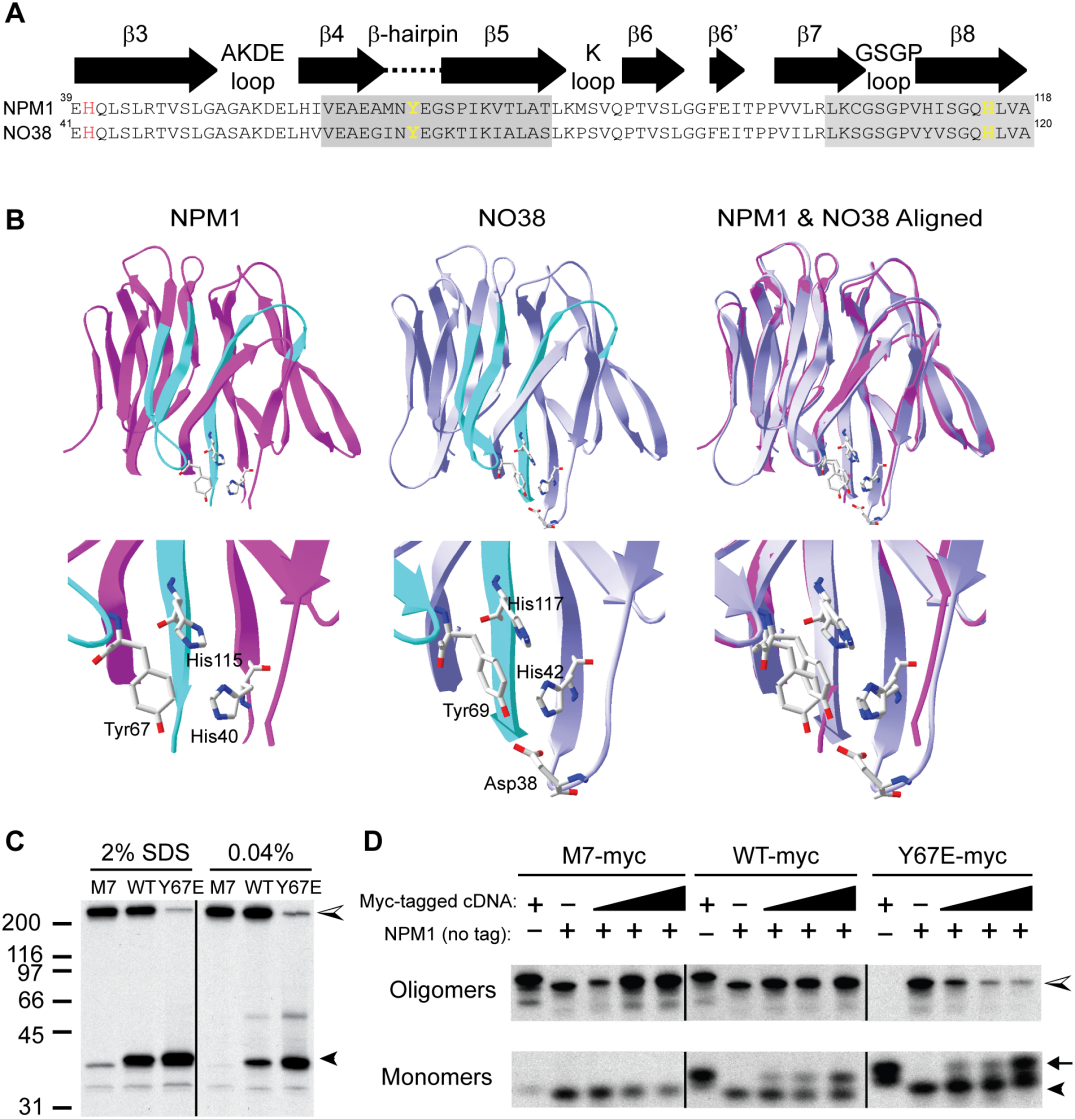
Tyrosine 67 is a critical residue at the β-hairpin “latch”–mutation to glutamate destabilizes oligomers. A) Structural alignment of NPM1 and NO38 showing β-strands 3–8, with bimodal deuterated region in grey and key residues highlighted in yellow and red. B) Ribbon structures of NPM1 and NO38 oligomer cores with 2 of 5 monomers shown, in order to highlight the key monomer-monomer interface. Areas with bimodal deuteration indicating EX1 kinetics are in aqua, corresponding regions (based on above alignment) in NO38 in same color. Key residues at the β-hairpin “latch” are labeled in the magnified images in the second row. C) 35S-methionine labeled IVTT product from M7-NPM (M7), wild-type NPM1 (WT) or Y67E-NPM (Y67E) cDNA, was mixed with sample buffer for final SDS concentration as noted; 2% SDS samples were boiled 5 minutes. All samples were separated by 10% SDS-PAGE and visualized by autoradiography. Monomers are indicated by filled arrowheads, oligomers by half-filled arrowheads. Black line indicates where intervening sample and blank lanes were cropped out to bring together relevant lanes. D) 35S-methionine labeled IVTT reactions with 50 ng of either untagged wild-type NPM1 (NPM1) or myc-tagged wild-type (WT-myc), M7-NPM (M7-myc), or Y67E-NPM (Y67E-myc) cDNA [first 2 lanes of each section]; mixtures of 50 ng untagged cDNA with 25, 50 or 100 ng myc-tagged cDNA are shown in last 3 lanes of each section. IVTT products were mixed with gel sample buffer to 2% SDS final but not boiled, then separated by 8% SDS-PAGE and visualized by autoradiography. Monomers of tagged (arrow) and untagged (arrowhead) nucleophosmin constructs migrate at distinct positions on SDS-PAGE, while oligomers increase in size as tagged constructs are incorporated (see M7-myc lanes, for example). Black lines indicate where intervening blank lanes were removed and sample lanes were reordered to better match data shown in (C).

For residues 60–118 in the oligomerization domain, M7-NPM peptides showed 10–20% deuteration, while multiple NPM1 peptides displayed bimodal spectra consistent with EX1 dynamics (peptides colored grey in Fig. 2A). Specifically, wild-type NPM1 peptides with bimodal deuteration localized primarily to one monomer-monomer interface formed by β-strand 4, the β-hairpin loop and β-strand 5 (residues 60–78) on one hand, and β-strand 8 (residues 109–115) on the adjacent subunit, as visualized in the X-ray crystal structure of human NPM1 (Figs. 2A and 3, [29]). Thus, our data indicate increased local accessibility of residues forming this interface in wild-type NPM1 oligomers, whereas this area in M7-NPM remained protected from deuteration.

### Mutation of tyrosine 67 at the β-hairpin loop interferes with oligomer formation

Because there were significant differences between deuteration patterns between wild-type NPM1 and M7-NPM at an interface including the β-hairpin loop, we hypothesized that this loop may itself be critical for oligomer stability. In order to further explore this question, we mutated tyrosine 67 (Y67), which is found at the tip of the β-hairpin loop in the X-ray crystal structure of human NPM1 oligomeric core [29] and also located in the middle of the first bimodal region in our DXMS analysis (Figs. 3A and 3B). From X-ray crystal models of both NPM1 [29] and NO38 [28], a *Xenopus* nucleolar chaperone with 70% identity in sequence, Y67 has key interactions with histidine 115 (H117 in NO38) and probably the short acidic tract (A1 in NLP family members) on the neighboring monomer (Fig. 3B). Although A1 was not visible in the human NPM1 structure, the X-ray diffraction model of NO38 showed that the corresponding tyrosine (Y69 in NO38) probably engages several acidic side chains, particularly aspartate 38 (D38). Based on this structural information, we predicted that a change to glutamic acid in NPM1, generating Y67E-NPM, would both remove favorable interactions with histidine residues and produce unfavorable contacts with the A1 acidic side groups, thus preventing closure of the β-hairpin “latch”.

In on previous work, we found that M7-NPM IVTT product would maintain stable oligomers with molecular weight greater than 200 kDa upon SDS-PAGE, even with 2% SDS in the sample buffer [32]. Here, we show that less than 10% of M7-NPM was found in the monomer form at 35 kDa under these standard SDS-PAGE conditions, while no monomer form was found when SDS was reduced to 0.04% (Fig. 3C). In contrast, wild-type NPM1 monomers were favored with 2% SDS, while the oligomer form was predominant with 0.04% SDS (Fig. 3C). In agreement with our hypothesized critical role for the β-hairpin “latch”, Y67E-NPM had minimal amounts of oligomer on SDS-PAGE under both standard and reduced SDS concentrations, with more than 95% of the IVTT product running as monomers at 35 kDa (Fig. 3C).

Next, we asked whether Y67E-NPM would interfere with the ability of wild-type NPM1 to form stable oligomers, since it should be able to interact with wild-type proteins except for the formation of a “latched” β-hairpin. When we proceeded to perform IVTT with wild-type NPM1 and myc-tagged Y67E-NPM, we found no incorporation of Y67E-NPM-myc into oligomers with wild-type NPM1 (Fig. 3D). Indeed, the oligomer band for co-IVTT of wild-type NPM1 and myc-tagged Y67E-NPM decreased with increasing amounts of Y67E-NPM-myc cDNA, while the monomer bands increased for both wild-type NPM1 and Y67E-NPM-myc (Fig. 3D). In contrast, myc-tagged M7-NPM or wild-type NPM1 proteins were easily incorporated into oligomers with untagged wild-type NPM1, leading to slower migration of the oligomer band as more tagged protein were incorporated into the complexes (Fig. 3D). Thus, mutation of one key residue at the β-hairpin “latch” was sufficient to prevent formation of SDS-stable nucleophosmin oligomers.

### NPM1 oligomer formation affects recognition and cleavage by granzyme B

**Figure 4:**
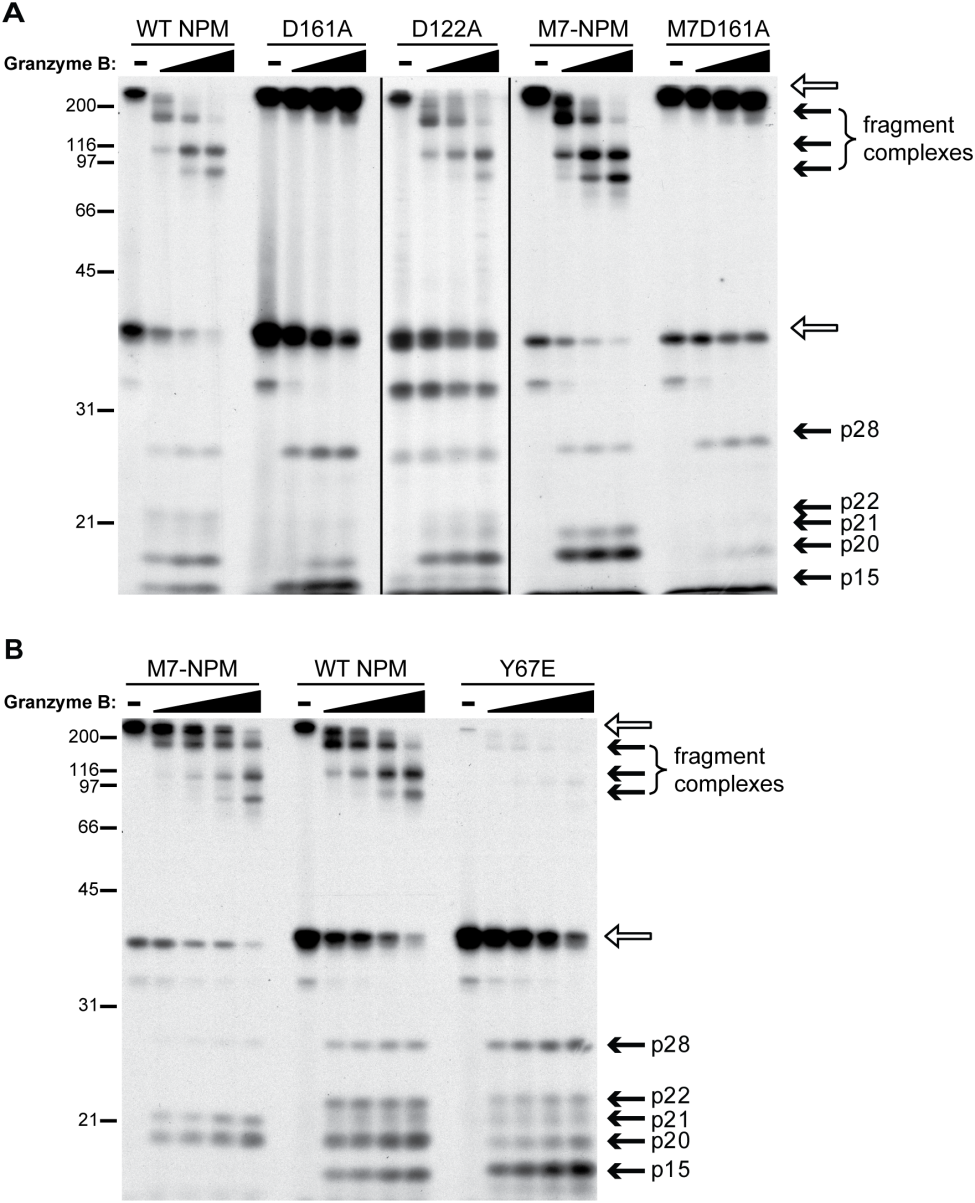
M7-NPM and Y67E-NPM produce distinct and complementary patterns of fragments upon granzyme B cleavage. A) 35S-methionine-labeled IVTT products (nucleophosmin construct indicated above lanes) were treated with increasing amounts of granzyme B (0, 27, 54, or 108 nM) for 60 minutes at 37°C and boiled in 2% SDS sample buffer before SDS-PAGE. Black lines indicate that D122A lanes were taken from a longer exposure of the same experiment, since there was less radioactive signal for this mutant. B) 35S-methionine-labeled IVTT products were incubated with granzyme B (0, 13.5, 27, 54, or 108 nM) for 60 minutes at 37°C before analysis as in (A). For both (A) and (B), open arrows denote intact oligomer and monomer, while solid arrows indicate fragments generated by granzyme B treatment.

Previously, Ulanet et al. [32] showed that increased oligomer formation by NPM1 in HCC tumor lysates correlated with alterations in cleavage by granzyme B, suggesting that conformational changes were affecting interactions with this highly specific serine protease. By both biochemical and DXMS analysis, M7-NPM preferentially formed a stabilized oligomer, and we wondered if this structural difference would also affect granzyme B cleavage patterns. As demonstrated before, wild-type NPM1 IVTT product is cleaved by granzyme B mainly at aspartate 122 and 161 (D122 and D161), generating distinct fragment patterns; targeted mutagenesis of these aspartate residues to alanine (D122A or D161A) resulted in a different subset of peptide fragments compared with wild-type NPM1 (Fig. 4A and [53]). Thus, cleavage at D122 generated peptides p28 and p15 (see D161A mutant, Fig. 4A), whereas cleavage at D161 produced a more complex pattern with individual fragments p20/p21/p22 and large complexes which likely represent some combination of intact and cleaved proteins (see D122A lanes in Fig. 4A). When we treated M7-NPM IVTT product with granzyme B, we found no formation of p15 and instead, increased signal for large fragment complexes and p20/p21, essentially replicating the pattern seen with the D122A mutant, though this cleavage site is unaltered in M7-NPM (Fig. 4A). To verify the preference of granzyme B for cleavage at D161 in M7-NPM, we mutated D161 to alanine and found that the mutant M7D161A was very resistant to granzyme B treatment, generating few fragments of any type (Fig. 4A, M7D161A lanes). Thus, enhanced oligomer formation by M7-NPM correlated with specific recognition and preferential cleavage by granzyme B at D161.

Notably, although our DXMS analysis showed that D122 is in an area readily accessible to solvent in both wild-type NPM1 and M7-NPM, the region immediately preceding this cleavage site formed part of the interface where wild-type NPM1 peptides showed EX1 deuteration patterns and M7-NPM peptides were poorly deuterated (Fig. 2A). Thus, disruption of the β-hairpin “latch” located within this interface may directly affect granzyme B binding and site selection, in addition to effects due to changes in oligomer stability. Indeed, granzyme B treatment of Y67E-NPM IVTT produced a fragment pattern distinct from both wild-type NPM1 and M7-NPM, with evidence of preferred cleavage at D122–p28 and p15 fragments were increased, and p20/p21/p22 and oligomeric fragment complexes were greatly reduced as a whole (Fig. 4B). Interestingly, the use of the preferred cleavage site in both M7-NPM and Y67E-NPM was not absolute, as demonstrated by the presence of small amounts of fragment associated with cleavage at the non-favored aspartate for each mutant form.

## Discussion

X-ray crystal structures have been solved for the oligomeric core of human and mouse NPM [29], [30], and likely represent models of the most stable and/or frequent conformations among a population of structures. The amino-terminal oligomerization domain is itself very stable, as demonstrated by intact folding at elevated temperatures (up to 90°C) and upon exposure to urea [54]. However, NPM1 oligomeric complexes have demonstrated sensitivity to ionic strength and denaturants [30], [52], [55]. In view of these past results, our DXMS studies have elucidated several interesting findings: 1) large areas in both wild-type NPM1 and M7-NPM were rapidly deuterated at 25°C, pH 7.9, and physiological salt concentrations, including hydrophobic monomer-monomer interfaces [29], [30]; 2) subtle structural differences between wild-type NPM1 and M7-NPM were evident under conditions of reduced deuteration kinetics and lowered ionic strength; and 3) only wild-type NPM1 showed bimodal deuteration patterns consistent with EX1 kinetics at a monomer-monomer interface including the β-hairpin loop, even under non-denaturing conditions.

Intriguingly, EX1 kinetics usually reflect important transitions or intermediates in protein folding. For example, these patterns were seen when conditions caused a progressively larger proportion of protein molecules to unfold, and also when proteins were allowed to refold after such a stress [39], [49], [56]. If individual domains of a multi-domain protein have varying stabilities, peptides from each region exhibit bimodal mass peaks under specific, differing conditions [49]. The presence of EX1 kinetics under non-denaturing conditions is extremely rare, but has been observed for a small set of SH3 domain proteins, including hematopoetic cell kinase (Hck) and Lyn kinases [57].

Our observation of bimodal deuteration spectra for multiple peptides comprising a specific monomer-monomer interface (β-strand 4, the β-hairpin loop, β-strand 5, and β-strand 8; Figs. 2 and 3) suggests that this specific region undergoes some degree of local “unfolding”. Moreover, we believe that the co-occurrence of wild-type NPM1 granzyme B cleavage fragments corresponding to use of both D161 and D122 supports our DXMS analysis that wild-type NPM1 undergoes dynamic structural shifts, likely between two distinct conformations which favor stabilized versus destabilized oligomers. However, it cannot be excluded that alternative initiation of translation occurred in wild-type NPM1 IVTT reactions, thus producing amino-terminally truncated constructs which contributed to the formation of SDS-stable oligomers and specific granzyme B cleavage fragments that match what were seen with M7-NPM IVTT reactions.

Although our DXMS studies used conditions thought to reduce deuteration kinetics [44] and destabilize NPM1 oligomers [30], [52], we believe that interactions at the monomer-monomer interface, including the β-hairpin loop, are important for NPM1 conformations under physiological conditions. In support of this, we first defined the critical role of the β-hairpin loop by changing a key residue, thus generating a mutant, Y67E-NPM, which could not form SDS-stable oligomers, and furthermore, prevented wild-type NPM1 oligomer formation in a dominant-negative fashion. Second, we found that granzyme B cleavage of wild-type NPM1 in the presence of 150 mM NaCl and at 37°C occurred at both D161 and D122, thereby producing fragment patterns which corresponded to granzyme B interactions with both stabilized and destabilized oligomers, as represented by M7-NPM and Y67E-NPM reaction products, respectively. Third, others have been able to disrupt oligomer formation in cells with a small molecule (NSC348884) designed to bind critical residues at the β-hairpin “latch”, specifically Y67, histidine 115 and histidine 29 ([58]). Finally, arginine-rich peptides derived from NPM1-binding proteins were also shown to interact with the β-hairpin “latch”, though resulting in oligomer stabilization in this case [30]. Thus, there is evidence that the β-hairpin “latch” is accessible to small molecules and peptides, with either disruptive or stabilizing effects on NPM1 oligomers, depending on the specific nature of binding interactions.

**Figure 5:**
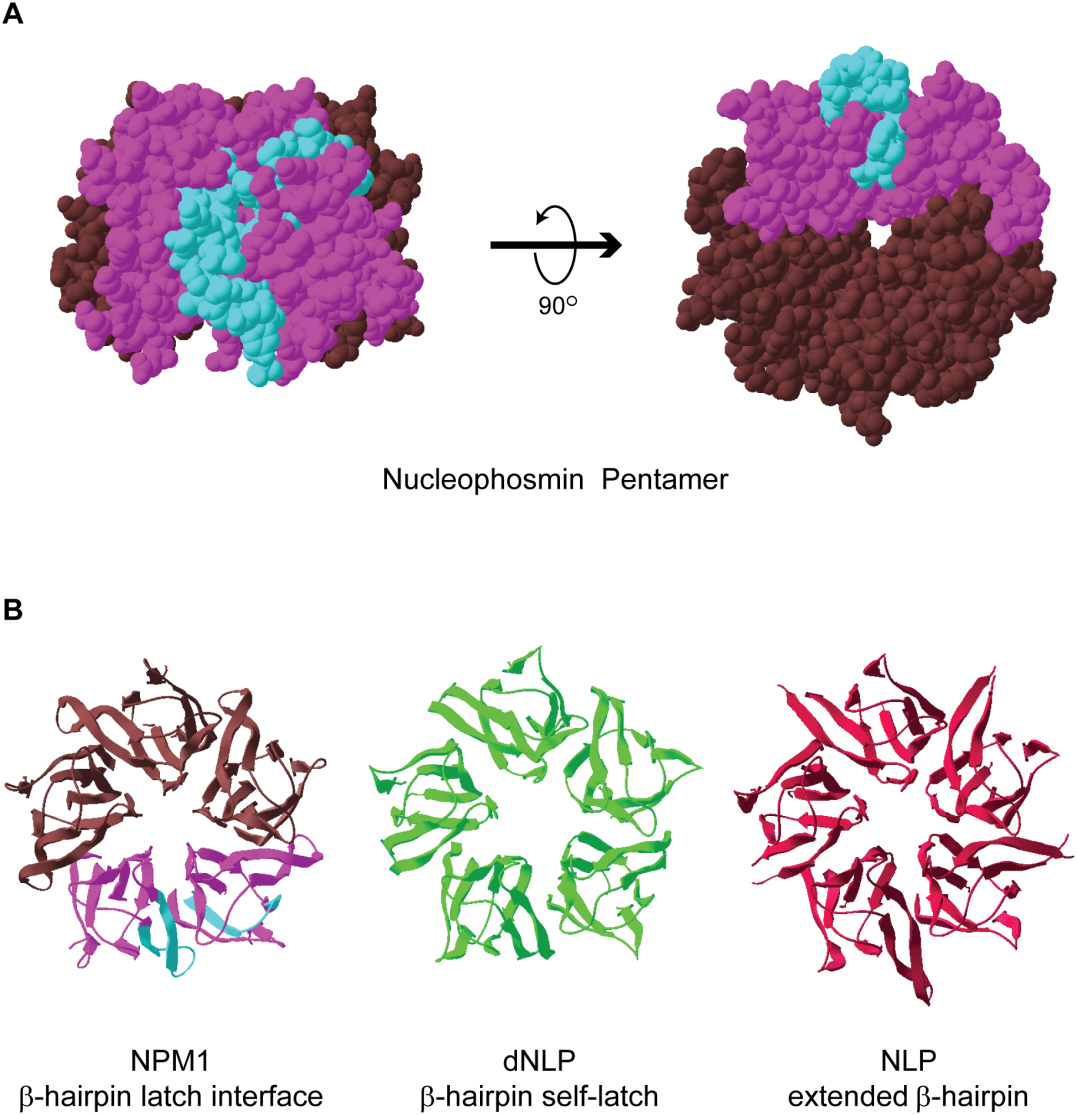
The β-hairpin loop area has significant structural differences between nucleoplasmin family members. A) Areas with bimodal deuteration (aqua) are depicted on two adjacent monomers (purple) in the X-ray crystal structure of NPM1 core pentamer (remaining monomers shown in brown). B) NPM1 core shown with same coloring scheme as in (A) is compared with Drosophila nucleoplasmin-like protein (dNLP, shown in green) and Xenopus nucleoplasmin (NLP, shown in maroon) core structures. All images prepared with Swiss PdbViewer (see Methods).

Additionally, the β-hairpin loop represents an area of key difference in the core crystal structures of NLP family members (Fig. 5). *Drosophila* nucleoplasmin-like protein (dNLP) has a “self-latch”, where the β-hairpin loop associates with residues in the same monomer [27], while the *Xenopus* NLP β-hairpin loop extends into the solvent, away from the core [26]. Only NPM1 and NO38 structures have shown the “latched” β-hairpin, and though nearly identical to each other in sequence, they are significantly different from other members of the NLP family in this region [28]–[30]. It is possible that the β-hairpin “latch” may be particularly critical for the nucleolar functions ascribed to NPM1 and NO38, in contrast with the chromatin chaperone activities demonstrated for NLP and dNLP.

Interestingly, disruption of the β-hairpin “latch” in wild-type NPM1 with NSC348884 led to increased apoptosis in various cancer cell lines [58], [59], suggesting that post-translational modifications in this area may have important functional consequences. Y67 was found to be a possible phosphorylation site during high throughput analysis of epidermal growth factor (EGF) stimulated HeLa cells [60], [61], and has also been predicted to be a kinase target by various *in* *silico* methods [62]. This tyrosine is highly conserved and predictions based on consensus target sequences have indicated several candidate kinases, including spleen tyrosine kinase (Syk), Src kinase, B lymphocyte kinase (BLK) and members of the mitogen activated protein kinase (MAPK) (see information for site 36 of NPM1 on PhosphoNET [63]). Additionally, tyrosine nitration due to oxidative signaling or stress is well described, and Y67 has favorable structural characteristics for this type of modification, including location within an exposed loop, following a polar residue (asparagine 66), and preceding an acidic amino acid (glutamate 68) [64]–[67].

It is possible that either phosphorylation or nitration of Y67 could be an early step in pathways which involve NPM1. By destabilizing NPM1 oligomers, such modifications may increase accessibility of other more protected phosphorylation targets within the pentamer, thus leading to greater or more permanent effects on NPM1 structure and function. This potentially important pathway for changing NPM1 function was highlighted in recent work by Mitrea et al. [30], who showed that an NPM1 mutant designed to mimic phosphorylation at a key serine residue (S48) had a decrease in oligomer stability concomitant with successful phosphorylation by protein kinase A (PKA). Because S48 is involved in an extensive hydrogen-bond network within the pentamer core, it was thought to be inaccessible without some initial destabilization of the oligomeric structure [30]. To fulfill this role, the authors proposed dual phosphorylation of two exposed residues, threonine 95 and serine 125; the simultaneous mutation of these to aspartic and glutamic acid, respectively, resulted in small amounts of phosphorylation by PKA, whereas the single point mutants remained unphosphorylated [30]. Modification of the β-hairpin loop was not explored in these studies, although our data indicate that this area can have profound effects on oligomer stability.

Additionally, our work demonstrates that loss of amino-terminal residues in NPM1 significantly impacted oligomer stability, corresponding with decreased accessibility at a specific monomer-monomer interface including the β-hairpin loop. A recently completed X-ray structural model of the mouse NPM1 oligomeric core showed that the amino-terminal residues, which include several large, negatively charged side-groups, were located on the same face of the oligomer as the β-hairpin loop and acidic residues of the A1 tract, thus contributing to a highly asymmetric distribution of charge [30]. It is possible that the amino-terminus acts to destabilize NPM1 oligomers, and its removal leads to pentamer stabilization. Also, the asymmetric distribution of charge may have amplified the destabilizing effect of mutating Y67 to a large, acidic residue, as occurred in Y67E-NPM.

We originally initiated our structural investigations of wild-type NPM1 and M7-NPM before there were systematic data available on alternate initiation of translation sites for mammalian proteins. Recently published studies on alternative initiation of translation in human and mouse cells have not found evidence of initiation at the seventh methionine for NPM1 [35]–[37], but it remains possible that M7-NPM occurs under certain circumstances, including during tumorigenesis. Additionally, M7-NPM oligomeric structure and biochemical behavior can provide an indication of what would be observed for constructs beginning at the fifth or ninth methionines. Our observations with M7-NPM may also be relevant for predicting the consequences of post-translational modifications at the amino-terminus. For example, serine 4 (S4) in wild-type NPM1 has been identified as a target of both polo-like kinase 1 (PLK1) [7] and a G protein-couple receptor kinase [68]; S4 phosphorylation was shown to be essential for mitotic progression in HeLa cells [7]. It is likely that attaching a phosphate group to S4 would impact the ability of this amino-terminal area to interact with other regions in NPM1, particularly given the asymmetric distribution of negative charge.

In preliminary studies to characterize the functional consequences associated with loss of the first six amino acids, we over-expressed M7-NPM in various human cell lines but did not observe differential subcellular localization of this mutant as compared with wild-type NPM1 (data not shown). We also undertook exploratory studies of the antigenic properties of M7-NPM, using immature dendritic cell lysosomal lysates to process recombinant protein samples; we found no significant differences in overall levels of degradation or specific peptide generation (data not shown). However, the impact of varying nucleophosmin oligomer stabilities on antigen processing may require proteases not found in these dendritic cell lysates and/or other interactions specific to the developing tumor cells and their microenvironment.

Previously, our group described biochemical and structural variability of NPM1 in tumor and differentiating cells, indicated by differences in epitope exposure, granzyme B recognition and resistance to SDS denaturation. In this current work, we undertook a detailed structural analysis of both wild-type NPM1 and M7-NPM under non-denaturing conditions, and found important differences at a specific monomer-monomer interface including the β-hairpin loop. We demonstrate here that interactions at the β-hairpin loop and the presence of the first six amino-terminal residues impacted oligomer formation and protein-protein interactions. It will be important to further understand which post-translational modifications may occur at these key areas and how these would affect the various roles attributed to NPM1, including its nucleolar chaperone and cell cycle functions.

## Supporting Information

S1 File **Detailed DXMS data for wild-type NPM1 and M7-NPM peptides at 25°C and 4°C.**
(XLSX)
